# Collagen Peptides in Urine: A New Promising Biomarker for the Detection of Colorectal Liver Metastases

**DOI:** 10.1371/journal.pone.0070918

**Published:** 2013-08-16

**Authors:** Mirelle E. E. Bröker, Zarina S. Lalmahomed, Henk P. Roest, Nick A. van Huizen, Lennard J. M. Dekker, Wim Calame, Cornelis Verhoef, Jan N. M. IJzermans, Theo M. Luider

**Affiliations:** 1 Department of Surgery, Division of Transplantation and Hepatobiliary Surgery, Erasmus University Medical Center, Rotterdam, The Netherlands; 2 StatistiCal BV, Wassenaar, The Netherlands; 3 Department of Neurology, Erasmus University Medical Center, Rotterdam, The Netherlands; The Chinese University of Hong Kong, Hong Kong

## Abstract

**Introduction:**

For both patients and the outpatient clinic the frequent follow-up visits after a resection of colorectal cancer (CRC) are time consuming and due to large patient numbers expensive. Therefore it is important to develop an effective non-invasive test for the detection of colorectal liver metastasis (CRLM) which could be used outside the hospital. The urine proteome is known to provide detailed information for monitoring changes in the physiology of humans. Urine collection is non-invasive and urine naturally occurring peptides (NOPs) have the advantage of being easily accessible without labour-intensive sample preparation. These advantages make it potentially useful for a quick and reliable application in clinical settings. In this study, we will focus on the identification and validation of urine NOPs to discriminate patients with CRLM from healthy controls.

**Materials and Methods:**

Urine samples were collected from 24 patients with CRLM and 25 healthy controls. In the first part of the study, samples were measured with a nano liquid chromatography (LC) system (Thermo Fisher Scientific, Germaring, Germany) coupled on-line to a hybrid linear ion trap/Orbitrap mass spectrometer (LTQ-Orbitrap-XL, Thermo Fisher Scientific, Bremen, Germany). A discovery set was used to construct the model and consecutively the validation set, being independent from the discovery set, to check the acquired model. From the peptides which were selected, multiple reaction monitoring (MRM's) were developed on a UPLC-MS/MS system.

**Results:**

Seven peptides were selected and applied in a discriminant analysis a sensitivity of 84.6% and a specificity of 92.3% were established (Canonical correlation:0.797, Eigenvalue:1.744, F:4.49, p:0.005). The peptides AGPP(-OH)GEAGKP(-OH)GEQGVP(-OH)GDLGA P(-OH)GP and KGNSGEP(-OH)GAPGSKGDTGAKGEP(-OH)GPVG were selected for further quantitative analysis which showed a sensitivity of 88% and a specificity of 88%.

**Conclusion:**

Urine proteomic analysis revealed two very promising peptides, both part from collagen type 1, AGPP(-OH)GEAGKP(-OH)GEQGVP(-OH)GDLGAP(-OH)GP and KGNSGEP(-OH)GAPGSKGDTGAKGEP(-OH)GPVG which could detect CRLM in a non-invasive manner.

## Introduction

Colorectal cancer (CRC) is the most common gastrointestinal malignancy worldwide and the 3^rd^ leading cause of cancer-related deaths in the western world. More than one-third of the patients develop colorectal liver metastases (CRLM) during the course of the disease, which are responsible for at least two-thirds of the deaths [1].

For the follow-up after CRC, blood level Carcinoembryonic antigen (CEA) is used to detect CRLM with a wide spread of sensitivity ranging from 58 to 89 percent [2], [3]. Because of this suboptimal sensitivity, liver imaging with ultrasonography and computer tomography are performed on a routine base. For both patients and the outpatient clinic the frequent follow-up visits are time consuming and due to large patient numbers expensive. Therefore it is important to develop an effective non-invasive test for the detection of CRLM which could be used outside the hospital.

Proteomic patterns in body fluids present new opportunities for the development of novel, highly sensitive diagnostic tools for detection of cancer [4], [5]. The urine proteome is known to provide detailed information for monitoring changes in the physiology of humans [5], [6]. Urine collection is non-invasive and urine naturally occurring peptides (NOPs) have the advantage of being easily accessible without labour-intensive sample preparation [7]. These advantages make it potentially useful for a quick and reliable application in clinical settings. To prove the concept it is possible to differentiate between different liver tumors (CRLM, Hepatocellular Carcinoma (HCC), Hepatocellular Adenoma) and not only measure peptides involved in the process of general tumor growth in the liver, we conducted a pilot-study. In the current study we demonstrated we could discriminate between these liver tumors with the use of peptides found in urine (unpublished work, poster presentation ESMO 2010). In this study, we will focus on the identification and validation of urine NOPs to discriminate patients with CRLM from healthy controls.

## Materials and Methods

### Ethics Statement

The use of patient materials was approved by the medical ethical committee of Erasmus Univercity Medical Center and written informed consent was obtained for all patients.

### Patient selection

We selected patients with Colorectal Liver metastasis (CRLM) and healthy kidney donors as controls. Inclusion criteria were; female gender, age above 18 years and written informed consent. The patients with CRLM underwent liver resection and their diagnoses were confirmed by the pathologist afterwards. Patients and controls were excluded if they were diagnosed with other malignancies or received prior chemotherapy.

A discovery set was formed of 23 patients that contained 11 patients with CRLM and 12 controls. In addition a validation set was formed with 26 patients, 13 with CRLM, and 13 controls.

### Sample collection

From patients with CRLM, 100 ml urine was collected (midstream morning urine of sober patients). Fifty ml of urine was sent to the chemical laboratory at room temperature for determination of standard parameters (e.g. creatinine, total urine protein). Aliquots of 10 ml were made from the remaining 50 ml urine and stored within 4 hours from sample withdrawal at −80°C.

### Sample preparation

Preparation of samples for proteomic analysis was performed as described previously [8] with some minor modifications. In brief, urine samples were thawed at room temperature and placed in a water bath for 30′at 30°C with regular mixing to dissolve the precipitate. At room temperature, 1.4 ml urine was centrifuged to remove remaining precipitate for 5′ at 2000 g. A volume of 1.2 ml of urine was diluted with 0.6 ml 3 M urea, 15 mM NH_4_OH, 0.03% (w/v) SDS solution. From this mixture, 1.5 ml high molecular weight components were discarded using Centrisart ultracentrifugation columns (Sartorius, Goettingen, Germany) with a molecular cut-off limit of 20 kDa at a centrifugal force of 2500 g for 10′. From this filtered sample 1.2 ml was applied to a PD-10 desalting column (GE Healthcare) equilibrated with 25 ml 0.01% NH_4_OH and allowed to completely enter the filter bed. To improve the yield of natural occurring peptides (NOPs), 1.3 ml of equilibration buffer was applied to the filter bed as a first step and allowed to wash out by gravity flow. Subsequently, 2 ml of equilibration buffer was applied to the PD-10 column, the flow-through was collected, lyophilized, and stored at +4°C until further use. Prior to analysis by the nano-liquid chromatography/mass spectrometry (LC-MS), samples were suspended in 50 µl of HPLC-grade H_2_O. For estimating the NOP content with the BCA assay (Pierce) 4 µL of sample was used. With the use of this information the sample was diluted to 0.8 µg peptide/µl to 0.1% trifluoroacetic acid/water for normalization.

### Qualitative Mass spectrometry analysis (Orbitrap)

In the first part of the study, samples were measured with a nano liquid chromatography (LC) system (Thermo Fisher Scientific, Germaring, Germany) coupled on-line to a hybrid linear ion trap/Orbitrap mass spectrometer (LTQ-Orbitrap-XL, Thermo Fisher Scientific, Bremen, Germany). Samples were loaded onto a trap column (PepMap C18, 300 μm ID 5 mm length, 5 μm particle size, 100 Å pore size; Thermo Fisher Scientific), washed and desalted for 10 minutes using 0.1% trifluoroacetic acid (TFA) (in water) as loading solvent. Next, the trap column was switched inline with the analytical column (PepMap C18, 75 μm ID ×250 mm, 3 μm particle and 100 Å pore size; Thermo Fisher Scientific) and peptides were eluted with the following binary gradient: starting with 100% solvent A, then from 0% to 25% solvent B in 60 min and from 25% to 50% solvent B in 30 min, where solvent A consisted of 2% acetonitrile and 0.1% formic acid (rest water), and solvent B consisted of 80% acetonitrile and 0.08% formic acid (rest water). All LC solvents were purchased at Biosolve, Valkenswaard, the Netherlands. Column flow rate was set at 300 nL/min. For electro-spray ionization (ESI), nano ESI emitters (New Objective, Woburn, MA, USA) were used and a spray voltage of 1.5 kV was applied. For Mass Spectrometry (MS) detection, a data-dependent acquisition method was used: high-resolution survey scan from 400–1800 Th. was detected in the Orbitrap (target of automatic gain control  = 10 E6, resolution  = 30,000 at 400 m/z, lock mass set to 445.120025 Th (protonated (Si(CH3)2O))6) [9]). On the basis of this full scan the five most intensive ions were consecutively isolated (AGC target set to 104 ions) and fragmented by collisional activated dissociation (applying 35% normalized collision energy) and detected in the ion trap. Precursor masses within a tolerance range of +/− 5 ppm that were selected once for MS/MS were excluded for MS/MS fragmentation for 3 minutes or until the precursor intensity fell below an S/N of 1.5 for more than five scans (early expiration). Orbitrap full scan spectra and ion trap MS/MS fragmentation spectra were acquired partially simultaneously.

### Data analysis

The MS/MS data from the raw data files of each sample were converted into mgf files using Extract-MSN (part of XCalibur version 2.0.7, Thermo Fisher Scientific Inc.) and used to perform database searches using Mascot (version 2.2.06; Matrix Science Inc., London, UK) against the human subset of the Uniprot-Swissprot database (version 2011–3, human taxonomy, 20,287 entries). For database searches the following parameters were used: oxidation as a variable modification of methionine, hydroxylation as a variable modification of proline and lysine, maximal missed cleavage of 0, and “none” was selected as enzyme. A peptide mass tolerance of 10 ppm and a MS/MS mass tolerance of 0.5 Da were accepted. An ion score of 25 was used as a cut-off value. Subsequently, the raw data files were loaded into the software package Progenesis LCMS (Version 2.5; Nonlineair Dynamics Ltd, New Castle, UK) and aligned for retention time., Only features with a charge state of +2 to+8 were included for further analyses. Next, the results of the Mascot database search were imported into Progenesis, and an export file was created in which for each individual sample the abundances of the detected features were displayed. Abundance levels of masses identified multiple times in one sample were summed into single abundance values prior to statistical analysis. To eliminate sporadic findings, identified masses not present in at least 3 samples of one group were subsequently excluded from further analysis.

### Statistical analysis

Statistical analyses on the patient characteristics were conducted in the Statistical Package for the Social Sciences (SPSS) version 20.0 (IBM Corp. Released 2011. IBM SPSS Statistics for Windows, Version 20.0. Armonk, NY: IBM Corp). Categorical variables are presented as number (percentage). Continuous variables are presented as median (range), Categorical variables were compared, after testing for normality, with the Chi-square test; continuous variables were compared with the Independent T-test test. A *p*-value <0.05 (two-sided) was considered significant.

Statistical analysis of the observations as obtained from mass spectrometry was performed using STATA (version 10, StataCorp, Texas, US). After testing for normality, using Shapiro-Wilks, univariate comparison between individuals from the groups was performed using Mann-Whitney U-test (parameter free) or unpaired t-test (parametric). This yielded many features (in raw abundance format) with significant outcome between the evaluated combinations. Subsequently, the identified features were analyzed using stepwise regression to construct a model containing a combination of those features with the highest sensitivity and specificity to distinguish the respective groups. This was done in combination with canonical linear discriminant analysis [10] to detect the various sensitivity and specificity details when the various models are applied.

Moreover background evaluation was also performed in which models were applied with at random features and outcome to compare the acquired sensitivity and specificity with those as obtained after statistical analysis.

The discovery set was used to construct the model, consecutively the validation set, being independent from the discovery set, to check the acquired model.

Throughout the study, applying two-sided testing, a significance level of 0.01 was considered to be statistically relevant.

The discovery set was subjected to a univariate analysis in order to identify masses significantly different (p<0.01) between CRLM patients and healthy controls that ought to be present in both discovery and validation. From the peptides with p-values <0.01 a best fit model was made in the discovery set and subsequently tested in the validation set to determine sensitivity and specificity.

### Quantitative mass spectrometry analysis (SRM)

For the peptides selected based on the statistical analysis heavy labeled stable isotopes were ordered (Pepscan, Lelystad, The Netherlands). A selective reaction monitoring (SRM) method for the selected peptides was developed and optimized on a UPLC which was online connected to a Xevo TQS mass spectrometer (Waters, Milford Massachusetts, USA).

The sample was trapped on a Symmetry C18 nanoACQUITY column (5 µm × 180 µm 20 mm) (Waters, Milford Massachusetts, USA) for 5 min and washed by a solution of 99% A and 1% B, solvent A 0.1% formic acid in water and solvent B is 0.1% formic acid in acetonitrile, with a flow of 8.00 µl/min. Followed by separation on an BEH 300 C18 column (1,7 µm * 75 µm * 200 mm) (7 µm ×75 µm ×15 cm) with a flow of 0.3 µl/min and a gradient starting with 98.5% A lowered in 30 min to 60%. In 0.10 min it was further decreased to 20% A and kept constant for 5 min following by an increase in 0.10 min, back to 98.5% for 20 min. Ions were produced by a Z-spray nanoflow source under atmospheric pressure using a capillary voltage of 3.00 kV, cone voltage of 50 V and a source offset of 50 V. The source temperature was maintained at 70°C. For every peptide 3 transitions were chosen differing in collision energy (table 1). Fragmentation is induced by collision dissociation with argon gas which is inserted with a flow rate of 0.15 ml/min. For the selection of the peptides for the final quantitative assay the following parameters were taken into account: No interference in used SRM transitions, co-elution of the peptide and internal standard (IS), linearity of response in measured concentration range, symmetry of peak shape and a signal intensity of at least 10 times the average observed background.

**Table 1:** The three transitions which have been developed for each peptide and the optimized collision energies.

Peptide	Parent mass (*m/z*)	Fragment (*m/z*)	Collision energy (V)
AGP P(-OH)GEAGK* P(-OH)GEQGV P(-OH)GDLGA P(-OH)GP	1088.51	527.28	37
		812.38	33
		1364.64	33
	1092.52	527.28	37
		812.38	33
		1372.66	33
K*GNSGEP(-OH)GAPGSK*GDTGAK*GEP(-OH)GPVG	786.04	442.23	29
		814.37	29
		957.94	25
	794.05	442.23	29
		822.38	29
		969.96	25

For each stable isotope labeled amino acid an extra mass of 8 Da is included.

*m/z* (mass-to-charge ratio); V (Voltage).

### Skyline

**Figure 1:**
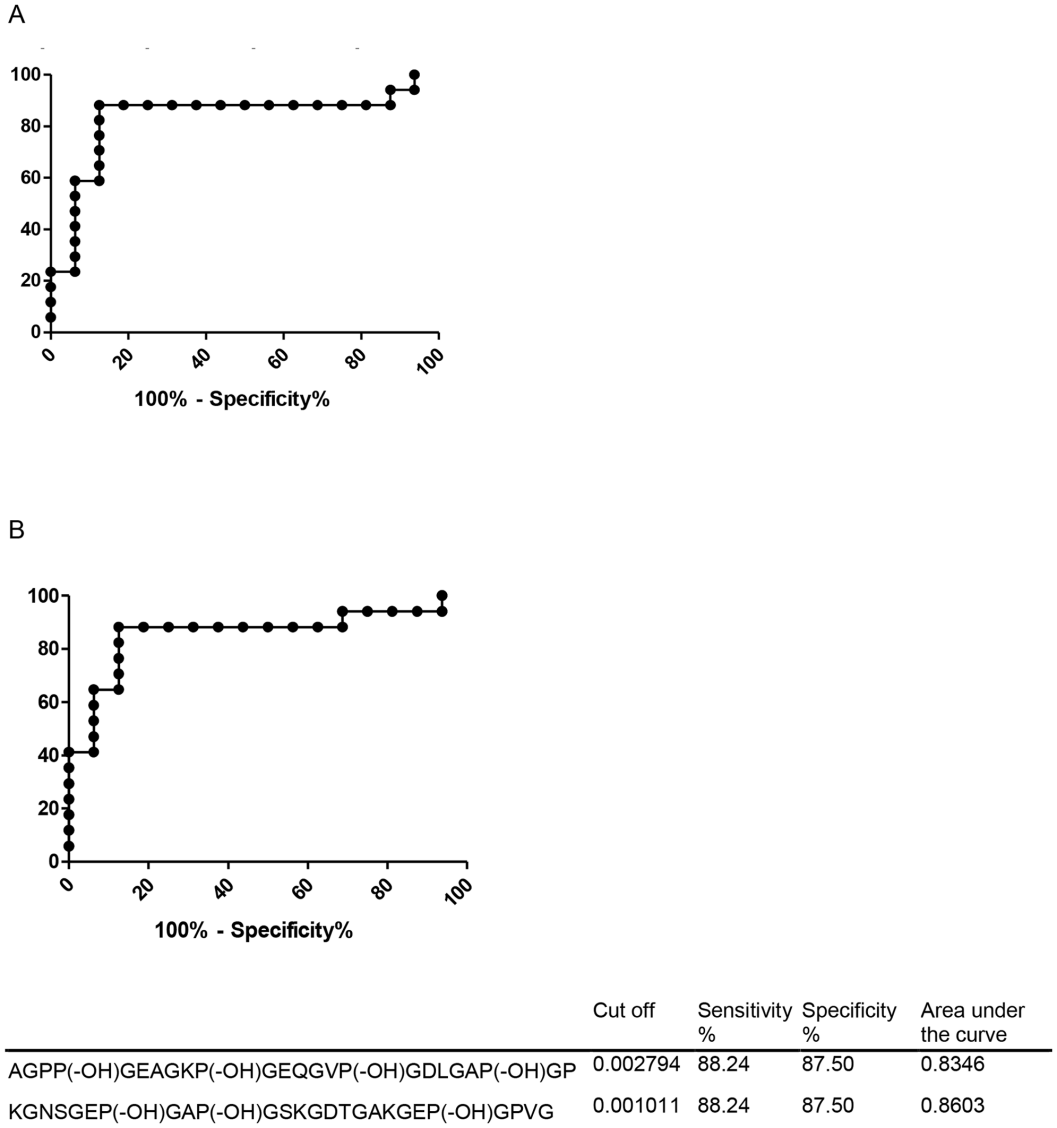
ROC-curves of the selected peptides AGPP(-OH)GEAGKP(-OH)GEQGVP(-OH)GDLGAP(-OH)GP and P(-OH)GNSGEP(-OH)GAP(-OH)GSKGDTGAKGEP(-OH)GPVG.

The following parameters were taken into account for the selection of the peptides that were used for the SRM assay: no interference in used SRM transition, co-elution of the peptide and its internal standard (IS), linearity of response in measured concentration range and the peak intensity should be at least 10 times above the background level. The peak analysis was done with Skyline v1.3.0.3871 (MacCoss Lab, University of Washington, WA, United States of America). The results exported from Skyline were further analyzed with Microsoft Excel2007 (Redmond, WA, United States of America) and GraphPad Prism v5.00 (GraphPad Software, San Diego California USA) A ROC-curve was plotted by GraphPad Prism v5.00 (GraphPad Software, San Diego California USA) (figure 1). The cut-off value was chosen whereby both sensitivity and specificity were as high as possible.

## Results

Patient characteristics and clinical chemistry data of the urines are presented in Table 2. A significant difference in age (*p* = 0.01) and Body Mass Index (BMI) (*p* = 0.04) is observed. Kidney function and urine protein were comparable between both groups.

**Table 2:** Basic patient characteristics.

Variable	CRLM (n = 24)	Healthy kidney donors (n = 25)	p- value
Age^1,3^	64 (43–81)	57 (34–75)	0.01
BMI^1,3^	23.6 (18–36)	27.3 (20–35)	0.04
No. of lesions^1^	2 (1–7) †	-	-
Size largest lesion (cm)^1^	3 (1–10) †	-	-
Serum creatinine >115 µM^2,4^	1† (4%)	0	0.29
Urine protein >0.14 g/L^2,4^	3 (12.5%)	0	0.07

† 1 missing

CRLM, Colorectal Liver Metastasis; BMI, Body Mass Index.

1 Data are presented as median with the range between brackets.

2 Data are presented as numbers with the percentage between brackets^.^

Data were analyzed using a ^3^Independent T-test or the ^4^Chi-squared test.

### Qualitative Mass spectrometry analysis (Orbitrap)

Of the 28830 and 57276 masses detected in the discovery and validation set, respectively, 2426 (8.6%) and 3424 (5.8%) unique peptide sequences were identified. These naturally occurring peptides belong to 189 and 445 proteins, respectively. Of the unique peptides identified 1386 (55%) derived from 26 different collagen proteins in the discovery set and 1303 (39%) from 34 in the validation set. Of all identified masses 80% of the abundant intensities is associated with collagen peptides. It occurred that same peptides were sequenced with one or more hydroxylated proline or lysine residues. Of all identified collagen peptides, 1702 peptides had 2 or more hydroxylations on either lysine or proline residues with a maximum of 11. These modified peptides are considered as unique peptides.

### Statistical analysis and regression modeling

Univariate analysis of the discovery set revealed 40 collagen peptides that were significantly different (p<0.01) between the healthy individuals and patients with CRLM.

A Multivariate analysis, using stepwise regression was applied in the discovery set to construct a relevant model using all identified peptides, showing significance between healthy individuals and CRLM patients. To obtain a 100% sensitivity and 100% specificity a 17-collagen peptides model was identified (Canonical correlation:0.9911, Eigenvalue:55.64, F:16.37, p:0.003) (Table 3).

**Table 3:** Sequences of the 17 Peptides used for the first model in the discovery set.

Mass (Da)	Sequence	Accesion code
2204.995	ADGQPGAKGEP(−OH)GDAGAKGDAGPP(−OH)GP	COL1A1
**2175.011**	**AGPP(−OH)GEAGKP(−OH)GEQGVP(−OH)GDLGAP(−OH)GP**	**COL1A1**
1927.909	DP(−OH)GETGEQGDRGIP(−OH)GHRG	COL1A1
1778.855	GAAGEP(−OH)GKAGERGVP(−OH)GPP(−OH)GA	COL1A1
2628.235	GLPGP(−OH)AGP(−OH)P(−OH)GEAGKP(−OH)GEQGVP(−OH)GDLGAP(−OH)GP	COL1A1
2561.128	GPP(−OH)GADGQP(−OH)GAP(−OH)GEP(−OH)GDAGAKGDAGP(−OH)PGP	COL1A1
2632.164	GPP(−OH)GADGQP(−OH)GAP(−OH)GEP(−OH)GDAGAKGDAGP(−OH)PGPA	COL1A1
2786.247	GPP(−OH)GADGQP(−OH)GAKGEP(−OH)GDAGAP(−OH)GDAGP(−OH)PGPAGP	COL1A1
2516.165	GPP(−OH)GKNGDDGEAGKP(−OH)GRP(−OH)GERGP(−OH)PGP	COL1A1
1734.781	GPP(−OH)GPP(−OH)GKNGDDGEAGKPG	COL1A1
1408.664	GPPGP(−OH)P(−OH)GP(−OH)PGPPGPPS	COL1A1
2371.086	P(−OH)GNSGEP(−OH)GAP(−OH)GSKGDTGAKGEP(−OH)GPVG	COL1A1
**2355.098**	**KGNSGEP(−OH)GAP(−OH)GSKGDTGAKGEP(−OH)GPVG**	**COL1A1**
1522.732	KP(−OH)GEQGVP(−OH)GDLGAP(−OH)GP	COL1A1
2989.483	NVGAP(−OH)GAKGARGSAGP(−OH)P(−OH)GATGFP(−OH)GAAGRVGPPGP(−OH)	COL1A1
2973.485	NVGAPGAP(−OH)GARGSAGPP(−OH)GATGFP(−OH)GAAGRVGP(−OH)PGP	COL1A1
2670.203	ERGEAGIP(−OH)GVP(−OH)GAP(−OH)GEDGKDGSP(−OH)GEP(−OH)GA	COL3A1

The amino acids which are underscored are hydroxylated. The two peptides which are written in bold are the finally selected peptides. (COL1A1 Collagen, type I, alpha 1).

Analysis of the samples from the validation set returned 58545 masses of which 3442 unique peptides could be identified. Out of these 3442 peptides, 1304 belonged to 34 different collagens.

Within the validation set, of the original 40 peptides identified in the discovery set as having a significant difference in raw abundance between both groups, 7 peptides could be identified. It was decided to use all 7 peptides to model the outcome based on the results in the validation set. When these 7 peptides were applied in a discriminant analysis a sensitivity of 84.6% and a specificity of 92.3% were established (Canonical correlation:0.797, Eigenvalue:1.744, F:4.49, p:0.005).

The peptides AGPP(-OH)GEAGK P(-OH)GEQGV P(-OH)GDLGA P(-OH)GP and KGNSGE P(-OH)GAPGSKGDTGAKGE P(-OH)GPVG were selected for further quantitative mass spectrometry analysis by selective reaction monitoring (SRM) based on a thorough evaluation on the criteria described in the materials and method section. The quantitative analysis resulted in a sensitivity of 88% and specificity of 89% (Figure 1).

These two peptides have been retested in the Orbitrap data showing a sensitivity and specificity of 72.7% and 100% in the discovery set and 69.2% and 84.6% in the validation set.

## Discussion

### Patients

For this study healthy kidney donors have been selected as a control group. These controls were selected because living kidney donors are examined intensively to exclude any disease. Because this specific control group was chosen, which were not matched with our CRLM-patients, a difference in patient characteristics in BMI (p = 0.04) and age (p = 0.01) was found. Only women were selected because the first pilotstudy we have performed included different solid liver tumors including HCA, a benign liver tumor which occurs very rarely in men [11].

In this study, a collagen profile has been discovered and validated in patients who were diagnosed with colorectal liver metastasis at the time of sampling. To identify the prognostic value of our profile, we will continue sampling during the regular follow-up in patients who underwent surgery because of CRC. Thereby patient variation presumably can be diminished and an even better sensitivity and specificity can be expected. The short coming of this study is the small number of patients. However we believe we describe a very innovative concept for the detection of colorectal liver metastasis. Furthermore the detection of different amounts of hydroxylated collagen type 1 may proof to be very valuable for clinical use and, in addition, it could contribute to the understanding of the liver seeding and homing of colorectal metastasis. Larger experiments need to be performed to demonstrate the efficacy of this approach.

### Peptide selection

In this study, only two stable isotope labelled peptides were technically suitable in our final analysis (table 1).Mischak et al. already showed data generated by different proteomics technologies are not always comparable [12]. Although the result could possibly be improved by taking more significant different peptides, this study already shows the new possibilities to use urine proteomic analysis to detect CRLM.

### Collagen

The two peptides identified in this study are part of collagen type 1. Collagens are macromolecular molecules which are eliminated due to secretion by the kidney. Type I collagen is the most abundant in stroma which is composed of the the extracellular matrix (ECM). In the adult liver the ECM is mostly composed of collagen type 1 and fibronectin [13], [14], [15], [16]. The structure of the ECM is not static, it remodels constantly as a consequence of development and disease [17]. This remodelling is a result of multiple processes that vary according to the initiating stimulus. The protein components of the ECM are cleaved by metalloproteinases (MMPs) and they seem to play a dominant role in this process of remodelling [18]. The remodelling of the ECM is an essential event before invasion of neoplastic cells into the stromal tissue and could explain our findings in the urine of patients with CRLM. The different expressions of type 1 collagen were also described in the stromal composition of tissue samples from CRC and CRLM [19]. The combination of these results provides evidence that type 1 collagen has a role in CRLM. However further research is needed to support these findings. Previously our research group identified tumour specific collagen-like peptides which are located in the blood vessels of brain tumours. These proteins are expressed in tumour blood vessels, but not in blood vessels of healthy brain tissue [20].

### Hydroxylation

Hydroxylation of peptides provides further stabilization or, depending on the location of the hydroxylation, the opposite, namely instability. Normally the hydroxylation in collagen sequences happens at the terminal residue in Gly–Pro–Pro repeats [21]. The final classifier existed of two hydroxylated collagen peptides with a sensitivity of 88%, a specificity of 88%. We hypothesize that in the liver the post-translational modification related to hydroxylation in collagen is altered due to the cancer cell invasion.

Although this study is based on relatively small numbers, urine proteomic analysis revealed two very promising peptides AGPP(-OH)GEAGK P(-OH)GEQGV P(-OH)GDLGA P(-OH)GP and KGNSGE P(-OH)GAPGSKGDTGAKGE P(-OH)GPVG, which are both part of collagen 1, to detect CRLM in a non-invasive manner.
